# Time-of-Day Effects on Post-Activation Potentiation Protocols: Effects of Different Tension Loads on Agility and Vertical Jump Performance in Judokas

**DOI:** 10.3390/medicina61030426

**Published:** 2025-02-28

**Authors:** Bilal Karakoç, Özgür Eken, Ahmet Kurtoğlu, Oğuzhan Arslan, İsmihan Eken, Safaa M. Elkholi

**Affiliations:** 1 Coaching Programme, Faculty of Sports Sciences, Yalova University, Yalova 7700, Türkiye; 2 Department of Physical Education and Sport Teaching, Faculty of Sports Sciences, Inonu University, Malatya 44280, Türkiye; 3 Department of Coaching, Faculty of Sport Science, Bandirma Onyedi Eylul University, Balikesir 10200, Türkiye; 4 Ministry of National Education, Ankara 01140, Türkiye; 5 Department of Physical Education and Sport Teaching, Firat University, Elazığ 23119, Türkiye; 6 Department of Rehabilitation Sciences, College of Health and Rehabilitation Sciences, Princess Nourah bint Abdulrahman University, P.O. Box 84428, Riyadh 11671, Saudi Arabia

**Keywords:** agility performance, combat sports, diurnal variation, judokas, post-activation potentiation, tension load, vertical jump

## Abstract

*Background and Objectives*: This study aimed to investigate the effects of different tension loads in post-activation potentiation protocols on agility and vertical jump performance across different times of day in trained judokas, addressing a significant gap in understanding the interaction between diurnal variations and post-activation potentiation protocol responses in combat sports. *Materials and Methods*: Seventeen male judokas (age: 21.41 ± 1.37 years) with ≥2 years of training experience participated in the study. Participants completed three different protocols: specific warm-up, the 80% post-activation potentiation protocol, and the 100% post-activation potentiation protocol, performed both in the morning (09:00–11:00) and evening (17:00–19:00) sessions. Performance was assessed using the Illinois Agility Test and countermovement jump. Protocols were randomized and counterbalanced over a 3-week period, with a minimum 48 h recovery between sessions. Statistical analysis employed repeated measures ANOVA (3 × 2) with Greenhouse–Geisser corrections. *Results*: Significant differences were observed in both protocols and time interactions for agility (F = 41.691, ηp^2^ = 0.864, *p* < 0.001; F = 23.893, ηp^2^ = 0.123, *p* < 0.001) and countermovement jump performance (F = 7.471, ηp^2^ = 0.410, *p* = 0.002; F = 38.651, ηp^2^ = 0.530, *p* < 0.001). The 80% post-activation potentiation protocol demonstrated superior performance outcomes compared to both specific warm-up and 100% post-activation potentiation protocols. Evening performances were generally better than morning performances for both agility and countermovement jump; however, the protocols/time interaction was not statistically significant (*p* > 0.05). *Conclusions*: The 80% post-activation potentiation protocol was most effective for enhancing both agility and vertical jump performance in judokas, with superior results observed during evening sessions. These findings provide valuable insights for optimizing warm-up strategies in judo competition, suggesting that lower-intensity post-activation potentiation protocols might be more beneficial than maximal loading, particularly during evening competitions.

## 1. Introduction

An effective warm-up plays a vital role in athletic performance beyond its physiological effects, influencing multiple mechanisms such as psychological readiness, injury prevention, and sport-specific movement preparation [1,2,3]. In combat sports like judo, where athletes must perform multiple high-intensity efforts throughout a competition day, optimizing warm-up strategies is crucial [4]. Psychological readiness is a key component, as cognitive focus, reaction time, and confidence directly impact performance outcomes [5,6]. Warm-up routines that incorporate visualization, attentional focus strategies, and sport-specific mental cues have been shown to enhance an athlete’s psychological preparedness, reducing anxiety and improving competitive engagement [7].

Additionally, injury prevention is a fundamental aspect of warm-up, as it facilitates progressive increases in muscle elasticity, joint mobility, and neuromuscular coordination [8,9]. Research suggests that dynamic warm-ups incorporating active stretching, mobility drills, and gradual load progression significantly reduce the risk of strains, sprains, and other soft tissue injuries [10,11]. Given the explosive and unpredictable movements in judo, a well-structured warm-up routine plays an instrumental role in minimizing injury risk [12].

Furthermore, sport-specific movement preparation ensures that athletes transition smoothly from general warm-up activities to movement patterns that closely replicate the demands of their sport [13]. For judokas, integrating sport-specific drills, such as dynamic gripping sequences, foot sweeps, and controlled takedown simulations, enhances neuromuscular readiness and optimizes technical execution in competition settings [14,15]. Within this framework, post-activation potentiation (PAP) represents a crucial physiological phenomenon in combat sports performance, defined as an acute enhancement in neuromuscular performance characteristics following maximal or near-maximal voluntary muscle contractions [16,17]. In the context of judo, where athletes must perform multiple high-intensity efforts throughout competition days, understanding the optimal implementation of PAP protocols becomes particularly relevant.

The underlying physiological mechanisms of PAP primarily involve myosin light chain phosphorylation following muscular stimulation [18,19]. During muscle contraction, calcium ions activate myosin light chain kinase through calcium–calmodulin interaction, leading to phosphorylation of myosin regulatory light chains [20]. This process increases the cross-bridge binding rate and enhances the calcium sensitivity of contractile proteins, ultimately improving muscle power generation potential [21,22].

Secondary mechanisms include enhanced motor unit recruitment through increased motor neuron excitability and alterations in muscle architecture, specifically reduced pennation angles that facilitate more efficient force transmission from muscle fibers to tendons [23,24,25]. Additionally, non-phosphorylation-dependent processes affecting Ca^2+^ sensitivity, such as increased muscle temperature, altered pH, enhanced blood flow, and increased muscle-tendon stiffness, contribute to the overall PAP response [26].

The effectiveness of PAP protocols is influenced by multiple variables, including exercise type, intensity, duration, rest intervals, and the athlete’s fitness level [27,28]. For judokas specifically, whose competitive success depends on explosive strength and agility, the optimization of these variables becomes crucial [29,30]. Similar to other sports [31], the requirement for multiple performances at different times of the day in judo competitions, coupled with the interaction of these demands with athletes’ circadian rhythms, underscores the critical importance of precise timing in the PAP.

Despite extensive research on PAP mechanisms and applications, there remains a significant gap in the literature regarding the interaction between diurnal variations and PAP responses in judo athletes, particularly concerning different tension loads and their effects on performance metrics. This study aimed to address this gap by investigating how different tension loads (100% 1 RM and 80% 6 RM) in PAP protocols affect agility and vertical jump performance across different times of day in trained judokas. The hypothesis of this study is ‘The effectiveness of PAP protocols will demonstrate significant diurnal variations, with enhanced performance outcomes during evening sessions compared to morning sessions’.

## 2. Materials and Methods

### 2.1. Participants

The study cohort consisted exclusively of first-degree black belt (1st Dan) judokas with active participation in national-level competitions. Participants were recruited during their off-competitive phase to eliminate potential confounding effects from competition preparation or rapid weight loss protocols. All subjects reported no history of sleep disorders or anxiety-related conditions. The study cohort comprised male judokas with a minimum of two years of competitive experience at the national level. All participants had maintained a consistent resistance training regimen (≥2 sessions/week) for at least 12 months prior to the investigation. Data collection commenced 14 days after the post-national championship competition to ensure adequate recovery and standardization. Prior to study initiation, participants were thoroughly briefed on the experimental protocols, potential risks, and study objectives. Written informed consent was obtained from all participants following a comprehensive explanation of the procedures. The investigation adhered to international ethical guidelines for chronobiological research in human subjects [32]. Standardized pre-assessment protocols were implemented to minimize confounding variables. Participants were instructed to maintain a minimum of eight hours of sleep prior to testing sessions and to consume their last meal at least two hours before morning and evening assessments. The effect size was determined based on the previous literature investigating post-activation potentiation (PAP) effects on agility and vertical jump performance in combat sports. Specifically, prior studies examining similar training protocols and their influence on explosive performance metrics reported effect sizes within a comparable range [27,31]. To ensure methodological rigor, we used G*Power 3.1.9.7 software to perform a priori power analysis. Given our research design, we selected an F-test with an effect size of 0.46, a significance level of α = 0.05, and statistical power (1 − β) of 0.80, which indicated a minimum required sample size of 15 participants. Since our study included 17 participants, we ensured that the sample was adequately powered to detect meaningful differences in performance variables across protocols and time-of-day conditions.

The study protocol was approved by the Institute’s Clinical Research Ethics Committee (Approval Number: 2023/4379). To ensure data quality and standardization, participants were required to abstain from high-intensity physical activity, alcohol consumption, and caffeine intake during the testing period. These dietary and lifestyle controls were systematically monitored throughout the experimental phase [33].

### 2.2. Methodology

All testing sessions, including both specific warm-up (SWU) and experimental protocols, were conducted in a standardized sequence during two distinct time periods: morning (09:00–11:00) and evening (17:00–19:00). Testing was performed at a temperature-controlled sports center facility under the supervision of a single investigator who was blinded to the participants’ protocol allocation. This standardized approach was implemented to minimize potential confounding variables and maintain methodological consistency throughout the study. To mitigate fatigue-induced confounding effects on test results, a minimum 48 h recovery period was implemented between the last training/competition session and the experimental protocols. Participants completed three distinct protocols: (1) SWU consisting of 10 min of metronome-guided exercises at 80 beats per minute (bpm) followed by 10 min of rest; (2) the same SWU protocol followed by an 80% PAP intervention; and (3) the SWU protocol followed by a 100% PAP intervention [31,34]. Regarding the justification for the selected intensity levels of 80% PAP and 100% PAP, previous studies have demonstrated that moderate-to-high loads (approximately 80% of 1 RM) are effective in eliciting PAP while minimizing fatigue [34]. This intensity has been shown to optimize neuromuscular excitation and subsequent performance improvements, particularly in explosive movements such as jumping and sprinting. Conversely, the inclusion of 100% PAP allows for comparison with maximal intensity conditioning contractions, which may induce greater potentiation effects but also carry a higher risk of fatigue [35]. By incorporating both intensities, our study aimed to assess the balance between potentiation and fatigue in athletic performance. Countermovement jump (CMJ) and agility performance were assessed following each protocol. The protocols were conducted at two different time intervals within the same day, with a minimum of 48 h between testing sessions to ensure complete recovery. All sessions were randomized and counterbalanced to minimize order effects. A standardized one-minute recovery interval was implemented between the countermovement jump (CMJ) and agility performance assessments. Each protocol maintained a consistent duration of 25 min. The protocols were administered in a randomized, counterbalanced order over a three-week period on consecutive days to control for potential order effects. All testing procedures were conducted in the athletes’ regular training and competition facilities to maintain ecological validity. For the PAP protocols, participants performed squat exercises at two different intensities: six repetitions maximum (6 RM) and one repetition maximum (1 RM). These loads were determined during the initial control session using the Brzycki (1993) formula and subsequently applied during the experimental sessions [36]. In this study, the one-repetition maximum (1 RM) was estimated following the methodology described by Brzycki (1993), using the formula: 1 RM = weight lifted/1.0278 − (0.0278 × repetitions). The 1 RM estimation was conducted during a dedicated familiarization session at least one week before the experimental trials [31,34] to ensure participants had sufficient recovery time before engaging in post-activation potentiation (PAP) protocols, thereby minimizing the risk of fatigue influencing their performance. To optimize the balance between potentiation and fatigue, the following time intervals were strictly maintained: (a) After completing the PAP protocol (80% and 100% of 1 RM), participants rested for exactly 5 min before performing the CMJ test. (b) Following the CMJ test, a 1 min rest interval was implemented before the Illinois Agility Test was conducted. These time intervals were selected based on previous research [31,34], which suggests that a 5 min recovery period optimally enhances PAP effects while preventing excessive fatigue. The 1 min interval before the Illinois Agility Test ensured that neuromuscular activation was maintained without inducing excessive fatigue between tests.

#### 2.2.1. Warm-Up Protocols

The Karvonen formula was used to calculate the heart rate reserve (HRR) in order to determine the running intensity of the judokas individually in the general warm-up phase before each test session. The warm-up intensity was defined by the target heart rate using the formula by Karvonen as follows: target heart rate  =  exercise intensity × (maximum heart rate − resting heart rate)  +  resting heart rate [37,38]. Following individual calculations of 50% Heart Rate Reserve (HRR), judokas performed a 5 min light jogging session under expert supervision [37]. Subsequently, judokas executed the SWU protocol comprising ten standardized exercises: foot sweeps, joint mobilization (wrist, finger, and ankle rotations), trunk mobility work (lateral stretches and rotator stretches), hip circles, knee flexion movements, bilateral cartwheels, and rolling sequences (forward, backward, and forward with leg abduction) (Table 1) [39]. They performed SWU consisting of 10 min of metronome-guided exercises at 80 beats per minute (bpm). The complete SWU protocol was conducted over a 10 min duration.

#### 2.2.2. Anthropometric Measurements

Anthropometric assessments were conducted using laboratory-grade calibrated instruments following standardized procedures (Tanita SC-330S, Amsterdam, The Netherlands) [40]. Body mass was quantified using a bioelectrical impedance analyzer (Tanita SC-330S, Amsterdam, The Netherlands) with a precision of 0.1 kg. Standing height was determined to the nearest 0.01 m utilizing a wall-mounted stadiometer (Seca Ltd., Bonn, Germany). Body composition indices, specifically body mass index (BMI) and lean-to-fat mass ratio, were evaluated using bioelectrical impedance analysis (Tanita SC-330S, Amsterdam, The Netherlands).

#### 2.2.3. Countermovement Jump

Countermovement jump (CMJ) performance was assessed using a force platform (Kistler, Winterthur, Switzerland) with data acquisition at 1000 Hz [41]. Participants executed the CMJ protocol from a standing position, performing a self-selected countermovement depth followed by an immediate vertical jump. To standardize the protocol, participants maintained hands-on-hips positioning throughout the movement, eliminating arm swing contribution. A qualified examiner ensured technical compliance, specifically monitoring for proper landing mechanics with no preliminary knee flexion. Maximum jump height (cm) was determined from two trials, separated by a 90 s recovery interval, with the superior performance recorded for analysis [42].

#### 2.2.4. Illinois Agility Test

The Illinois Agility Test is not entirely judo-specific; it serves as a general measure of agility, providing a basis for comparing performance improvements across different warm-up and PAP protocols. The Illinois Agility Test, validated for its psychometric properties in assessing multi-directional speed and change-of-direction ability, was administered within a standardized 10 m × 5 m testing zone. The protocol utilized four centrally positioned markers, with participants executing prescribed directional changes through a standardized course configuration. This assessment has demonstrated high test–retest reliability and construct validity for evaluating agility performance in athletic populations [43].

#### 2.2.5. Statistical Analysis

All statistical analyses were performed using Python 3, 3.9 (PSF, Amsterdam, The Netherlands). The Shapiro–Wilk test was used to analyze the normality of the data. The repeated measures ANOVA (3 × 2) test was performed to analyze the data of three different protocols (SWU, 80% PAP, and 100% PAP) at 2 different times (morning and evening). These analyses were performed with the AnovaRM function. Greenhouse–Geisser corrections were applied because the sphericity assumption was not verified. The Tukey HSD post hoc test was used to determine the difference between groups as a result of the ANOVA test. Statsmodels, scipy, matplotlib, and seaborn libraries were used to visualize the data. In addition, partial eta squared (ηp^2^) values were analyzed for effect sizes. The significance level was determined as 0.05.

## 3. Results

Table 2 presents the descriptive statistics of the demographic data of the participants. The study included 17 participants aged between 20 and 24 years, with a mean age of 21.41 ± 1.37 years. The mean values for height, body mass, BMI, %100 1 RM squat, and %80 6 RM squat were 179.35 ± 5.60 cm, 74.35 ± 7.85 kg, 23.07 ± 1.64, 56.17 ± 11.45 kg, and 48.88 ± 9.81 kg, respectively.

**Figure 1:**
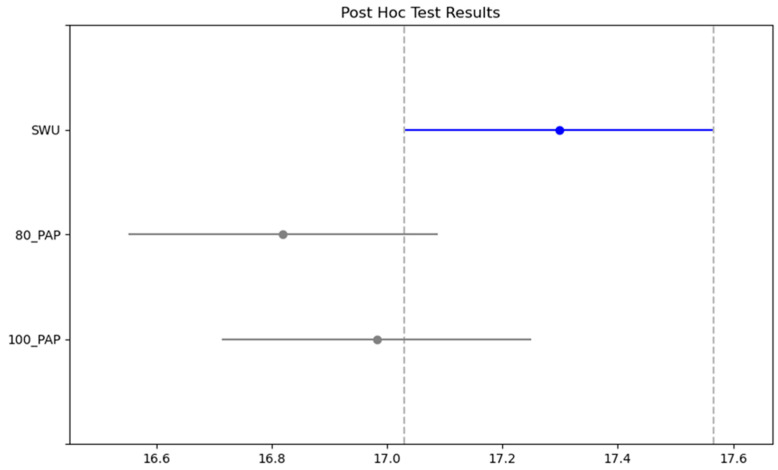
Post hoc results of the Illinois test between protocols.

**Figure 2:**
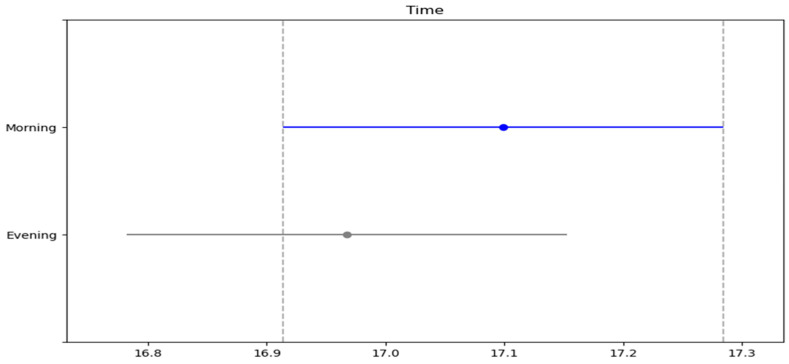
Post hoc test results of Illinois test between time.

In Table 3, the measured parameters of the participants for the Illinois test were compared. Accordingly, there was a significant difference in protocols (SWU, 80% PAP and 100% PAP) and time interaction (morning, evening) (F = 41.691, ηp^2^ = 0.864, *p* < 0.001; F = 23.893, ηp^2^ = 0.123, *p* < 0.001, respectively). No significant difference was detected between the protocols/time interaction of protocols and time (*p* > 0.05). In the posthoc test, the Illinois test results were lowest in the SWU and best in the 80% PAP protocol (Figure 1). In addition, although there was no significant difference, the Illinois performance was better in the evening (Figure 2).

**Figure 3:**
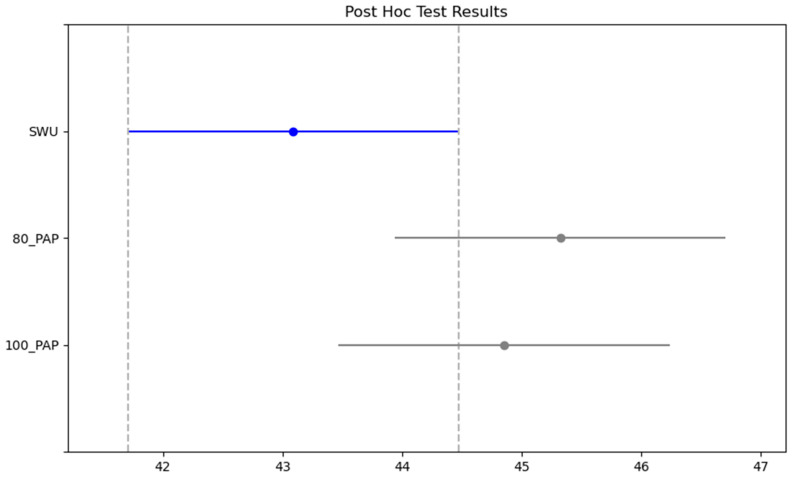
Posthoc results of the CMJ test between protocols.

**Figure 4:**
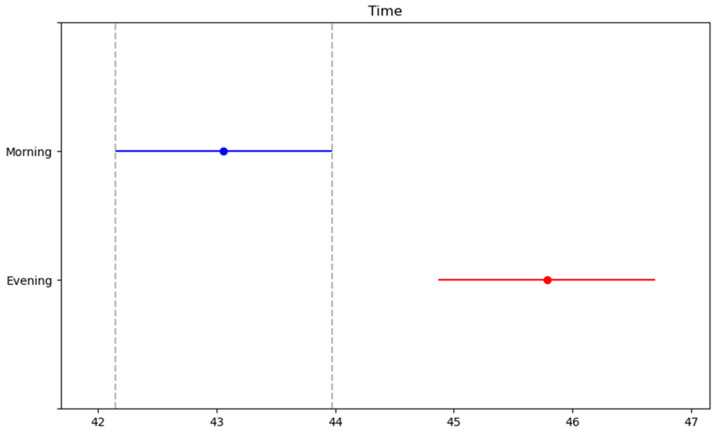
Post hoc test results of CMJ test between time.

In Table 4, the measured parameters of the participants for the Illinois test were compared. Accordingly, there was a significant difference in protocols (SWU, 80% PAP and 100% PAP) and time interaction (morning, evening) (F = 7.471, η^2^p = 0.410, *p* = 0.002; F = 38.651, η^2^p = 0.530, *p* < 0.001, respectively). No significant difference was detected between the protocols/time interaction of protocols and time (*p* > 0.05). In the posthoc test, the CMJ test results were lowest in SWU and best in the 80% PAP protocol (Figure 3). In addition, the CMJ performance was better in the evening (Figure 4).

## 4. Discussion

The primary aim of this investigation was to examine the effects of different PAP protocols on agility and vertical jump performance across different times of day in trained judokas. Our findings revealed several key insights that contribute to the understanding of optimal performance enhancement strategies in combat sports.

The superior performance outcomes observed with the 80% PAP protocol compared to both the 100% PAP and SWU protocols align with previous research suggesting that submaximal loads may be more effective for inducing PAP responses [31,34]. This finding supports the work of Wilson et al. (2013), who demonstrated that moderate-intensity protocols (60–84% 1 RM) generally produce superior performance enhancements compared to higher-intensity protocols [27]. The reduced effectiveness of the 100% PAP protocol might be attributed to excessive fatigue accumulation, which potentially masked the potentiation effects, as previously suggested by Tillin and Bishop (2009) [35].

The enhanced agility performance following the 80% PAP protocol is particularly noteworthy, as it demonstrates the transfer of PAP effects to complex, multi-directional movements. This finding extends previous research that primarily focused on linear sprint performance or vertical jump height [44,45,46]. The improvement in agility performance may be attributed to enhanced neuromuscular activation and motor unit recruitment patterns [47,48,49]. However, when compared with a similar study, it was concluded that the difference between 80% PAP and other parameters in our study was not very high [50]. The main reason for this situation is thought to be the type of sport (individual sport, team sport) because the frequent repetition of agility and CMJ performance in the competition may naturally increase these performances in team sports.

The observed superior evening performance across all protocols aligns with established chronobiological research indicating peak physical performance during the late afternoon and early evening hours [39,50,51]. The potential mechanisms underlying better evening performance can be attributed to several physiological and neuromuscular factors influenced by circadian rhythms. Research suggests that body temperature, which tends to peak in the evening, plays a critical role in enhancing muscle function, flexibility, and enzymatic activity, thereby improving physical performance [52,53]. PAP effects, which enhance muscle contractile properties through mechanisms like myosin light chain phosphorylation and fast-twitch fiber recruitment, could be more pronounced in the evening when fatigue is lower and recovery from prior activities is optimized [31,54]. These combined factors likely explain the observed improvements in athletic performance during evening hours. Our findings extend this understanding by demonstrating that the effectiveness of PAP protocols is influenced by circadian rhythms, with evening sessions consistently producing better outcomes. This temporal optimization of performance could be attributed to higher core body temperature and enhanced neuromuscular function during evening hours, as suggested by previous studies [4,55,56].

The differential responses to varying PAP intensities across different times of day have significant implications for judo competition preparation. The superior results achieved with the 80% PAP protocol, particularly during evening sessions, suggest that coaches and athletes should consider both the timing of competition and the intensity of warm-up protocols when designing pre-competition routines. This finding is especially relevant given that many judo competitions extend throughout the day, requiring athletes to perform optimally at various times.

The enhanced performance observed with the 80% PAP protocol may be explained by several physiological mechanisms. First, the moderate intensity likely achieved an optimal balance between potentiation and fatigue [57,58]. Second, the protocol may have enhanced calcium sensitivity and myosin light chain phosphorylation without excessive metabolic cost [59,60,61]. The reduced effectiveness of the 100% PAP protocol supports the notion that excessive loading may compromise the potentiation–fatigue relationship, particularly in sports requiring complex motor skills like judo.

While this study focused on judo, PAP is a well-established mechanism in various sports requiring explosive strength and agility [62,63,64]. In sports such as wrestling, taekwondo, and mixed martial arts, explosive movements are crucial for performance, making PAP strategies highly relevant [65,66]. Research in basketball and soccer has also demonstrated the benefits of PAP in enhancing sprint and jump performance [67,68], suggesting that findings from combat sports can be extended to other athletic domains. Future studies should explore how PAP strategies vary across different sports and whether similar submaximal load protocols (such as 80% of 1 RM) yield consistent performance improvements across disciplines.

While this study provides valuable insights, several limitations should be acknowledged. First, the participant pool was limited to male judokas, potentially limiting generalizability to female athletes. Future research should examine gender-specific responses to PAP protocols across different times of day. Second, while we controlled for training status, individual variations in fiber-type distribution and strength levels might influence PAP responses. Future studies could incorporate muscle biopsies or advanced imaging techniques to better understand the underlying physiological mechanisms. An important limitation of our study is that the participants belong to a single branch. This may be due to the fact that branch-specific technical skills and general physical fitness parameters may be improved in some and not in others. It is thought that studies comparing different sports branches are also needed. As a result of this situation, the limitations of the agility test in assessing judo-specific agility, it is suggested that future studies should include alternative agility tests adapted to judo. In particular, we suggest the use of tests that better reflect the dynamic demands of judo, such as the Uchikomi Agility Test, specific judo fitness test, Re-active Agility Test, or modified hexagonal agility tests.

Additionally, investigating the durability of PAP effects throughout a typical competition day and examining how different recovery periods between PAP protocols and performance might influence outcomes would provide valuable practical insights. Future research might also consider examining these protocols’ effects on sport-specific technical movements in judo.

Future research should explore the underlying physiological mechanisms contributing to the enhanced performance observed with submaximal loads and investigate the applicability of these findings to female judokas and athletes from other combat sports. Additionally, examining the long-term effects of incorporating these PAP protocols into regular training routines could provide further insights into their practical applications.

## 5. Conclusions

This study investigated the effects of different tension loads in PAP protocols on agility and vertical jump performance across different times of day in trained judokas. The findings demonstrated that the 80% PAP protocol was most effective in enhancing both agility and vertical jump performance compared to the 100% PAP and SWU protocols. Additionally, performances were generally better during evening sessions than morning sessions, highlighting the influence of diurnal variations on physical performance. These results have significant implications for optimizing warm-up strategies in judo and potentially other combat sports. Implementing submaximal tension loads in PAP protocols, particularly during evening sessions, may enhance athletes’ agility and explosive strength, leading to improved competitive performance. Coaches and athletes should consider both the intensity of PAP protocols and the timing of training sessions to maximize performance outcomes.

## Figures and Tables

**Table 1:** SWU Protocols.

Exercise Component	Description	Duration/Repetitions	Intensity
Foot Sweeps	Dynamic sweeping movements simulating judo techniques	30 s work–30 s rest	50% HRR
Joint Mobilization	Wrist rotations, finger flexion/extension, ankle circles	30 s work–30 s rest	50% HRR
Trunk Lateral Stretches	Alternating side bends with controlled movement	30 s work–30 s rest	50% HRR
Trunk Rotator Stretches	Dynamic rotational movements of the torso	30 s work–30 s rest	50% HRR
Hip Circles	Full-range circular hip movements in both directions	30 s work–30 s rest	50% HRR
Knee Flexion Movements	Controlled knee bends with proper alignment	30 s work–30 s rest	50% HRR
Bilateral Cartwheels	Cartwheels performed alternating both sides	30 s work–30 s rest	50% HRR
Forward Rolls	Standard forward rolling technique	30 s work–30 s rest	50% HRR
Backward Rolls	Standard backward rolling technique	30 s work–30 s rest	50% HRR
Forward Rolls with Leg Abduction	Forward rolls with legs spread during execution	30 s work–30 s rest	50% HRR

**Table 2:** Descriptive statistics on the demographic data of the participants.

Variable	N	Mean ± SD	Minimum	Maximum
Height (cm)	17	179.35 ± 5.60	170.0	190.0
Body mass (kg)		74.35 ± 7.85	62.0	97.0
Age (years)		21.41 ± 1.37	20.0	24.0
BMI (kg/m^2^)		23.07 ± 1.64	21.1	26.9
% 100 1 RM squat (kg)		56.17 ± 11.45	38.0	77.0
% 80 6 RM squat (kg)		48.88 ± 9.81	33.0	67.0

BMI: Body mass index, Mean: mean values, SD: standard deviation, RM: repeat maximum.

**Table 3:** Comparison of measured values of Illinois Test.

Groups	Mean ± SD	Between Protocols F ηp^2^ *p*-Value	Time F ηp^2^ *p*-Value	Protocols/Time Interaction
SWU-M	17.34 ± 0.99	F = 41.691 η^2^p = 0.864 *p* < 0.001	F = 23.893 η^2^p = 0.123 *p* < 0.001	F = 0.568 η^2^p = 0.011 *p* = 0.571
SWU-E	17.25 ± 1.07			
%80 PAP-M	16.90 ± 0.87			
%80 PAP-E	16.73 ± 0.88			
%100 PAP-M	17.04 ± 0.91			
%100 PAP-E	16.92 ± 0.88			

SWU-M: specific warm-up-morning, SWU-E: specific warm-up-evening, %80 PAP-M: %80 post-activation potentiation-morning, %80 PAP-E: %80 post-activation potentiation-evening, %100 PAP-M: %100 post-activation potentiation-morning, %100 PAP-E: %100 post-activation potentiation-evening.

**Table 4:** Comparison of measured values of CJM performance.

Groups	Mean ± SD	Between Protocols F η^2^p *p*-Value	Time F η^2^p *p*-Value	Protocols/Time Interaction
SWU-M	41.47 ± 4.55	F = 7.471 η^2^p = 0.410 *p* = 0.002	F = 38.651 η^2^p = 0.530 *p* < 0.001	F = 0.568 η^2^p = 0.058 *p* = 0.356
SWU-E	44.70 ± 5.58			
%80 PAP-M	43.94 ± 3.99			
%80 PAP-E	46.70 ± 4.38			
%100 PAP-M	43.76 ± 4.11			
%100 PAP-E	45.94 ± 5.08			

SWU-M: specific warm-up-morning, SWU-E: specific warm-up-evening, %80 PAP-M: %80 post-activation potentiation-morning, %80 PAP-E: %80 post-activation potentiation-evening, %100 PAP-M: %100 post-activation potentiation-morning, %100 PAP-E: %100 post-activation potentiation-evening.

## Data Availability

The datasets generated and/or analyzed during the current research are available from the corresponding author upon reasonable request.

## Abbreviations

The following abbreviations are used in this manuscript:

PAP	Multidisciplinary Digital Publishing Institute
SWU	specific warm-up
CMJ	countermovement jump
RM	repetition maximum
HRR	Heart Rate Reserve
BMI	body mass index
η2p	partial eta squared
